# Combining Primed Photoconversion and UV-Photoactivation for Aberration-Free, Live-Cell Compliant Multi-Color Single-Molecule Localization Microscopy Imaging

**DOI:** 10.3390/ijms18071524

**Published:** 2017-07-14

**Authors:** David Virant, Bartosz Turkowyd, Alexander Balinovic, Ulrike Endesfelder

**Affiliations:** Department of Systems and Synthetic Microbiology, Max Planck Institute for Terrestrial Microbiology & LOEWE Center for Synthetic Microbiology (SYNMIKRO), Karl-von-Frisch-Str. 16, 35043 Marburg, Germany; david.virant@synmikro.mpi-marburg.mpg.de (D.V.); bartosz.turkowyd@synmikro.mpi-marburg.mpg.de (B.T.); alexander.balinovic@synmikro.mpi-marburg.mpg.de (A.B.)

**Keywords:** multi-color imaging, primed conversion, live cell imaging, single-molecule localization microscopy

## Abstract

Super-resolution fluorescence microscopy plays a major role in revealing the organization and dynamics of living cells. Nevertheless, single-molecule localization microscopy imaging of multiple targets is still limited by the availability of suitable fluorophore combinations. Here, we introduce a novel imaging strategy which combines primed photoconversion (PC) and UV-photoactivation for imaging different molecular species tagged by suitable fluorescent protein combinations. In this approach, the fluorescent proteins can be specifically photoactivated/-converted by different light wavelengths using PC and UV-activation modes but emit fluorescence in the same spectral emission channel. We demonstrate that this aberration-free, live-cell compatible imaging method can be applied to various targets in bacteria, yeast and mammalian cells and can be advantageously combined with correlative imaging schemes.

## 1. Introduction

Over the past decade, rapid advances in single-molecule localization microscopy (SMLM) techniques have created a large, quantitative imaging toolbox which allows for the direct observations of molecular processes of life at the nanometer scale [1,2,3]. Part of its tremendous impact is owed to the fact that, just like in conventional fluorescence microscopy, individual species of molecules can be visualized and placed into context to each other by specific fluorescence tags in situ as well as in vivo.

Nowadays, multi-color SMLM imaging is frequently performed. However, while several approaches for multi-color SMLM imaging in fixed cells have been optimized for ideal combinations of bright fluorophores with complementary staining (e.g., antibody, nanobody or protein-tag stainings), similar photoswitching requirements and distinct read-out, the choice of fluorophore combinations for multi-color imaging of difficult-to-access or densely packed samples or under physiological conditions and in living cells remains challenging [3,4,5]. Especially in live-cell applications the choice of suitable fluorophore pairs is limited: apart from a small number of membrane-permeable, fluorescent dyes, which photoswitch in cellular environments without the need of further imaging buffers (e.g., tetramethylrhodamine (TMR) or Atto655 [3] or the recently developed paJF549 [6]), the most popular fluorophores are genetically encoded, photoactivatable or photoconvertible fluorescent proteins (pa- and pcFPs) like the widespread Anthozoan green-to-red pcFPs (e.g., the Eos, Dendra, Maple and Dronpa families) and DsRed derived dark-to-red paFPs (e.g., PAmCherry, PAmKate and PAtagRFPs) [1,4,5,7]. Despite being popular in quantitative imaging and single-particle tracking approaches [2,5,8], the simultaneous use of multiple pcFPs and paFPs is largely precluded by their overlapping absorption and emission spectra: they are photoactivated/-converted by illumination in the near UV light range and a vast majority of common FPs with desirable properties (being bright and monomeric, exhibiting robust photophysics in varying redox-environment and a quantitative read-out [1,4,5]) emit fluorescence in a narrow, orange-red emission channel with only a few alternative FPs in other color channels, e.g., the green fluorescing paGFP [9], psCFP2 and Dronpa [10], or the red fluorescing psmOrange [11], which have been used for dual-color SMLM.

By utilizing the recently developed photoconversion technique of primed conversion (PC) for green-to-red pcFPs, we here introduce a new dual-color strategy. PC, which was first identified and characterized for Dendra2 [12,13] and has recently been shown to be very efficient for green-to-red pcFPs with threonine at residue 69, makes use of a combined illumination of 488 nm and near-infrared light in the range 700–800 nm instead of the common UV-photoconversion to convert common pcFPs from their green to their red form [12,14]. paFPs, on the other hand, only absorb light in the ultraviolet range prior to activation. As such, the 488 nm light used for PC should have no effect on the paFP. We propose that this fact could be advantageously exploited in a dual-phase, dual-color imaging scheme with a single fluorescence read-out channel. First, a PC-suitable pcFP, e.g., Dendra2 [12,13,14,15] or mEos3.2-A69T [14], is imaged using a combination of 488 and 730 nm light for photoconversion. After all pcFPs have been read out, the still-intact paFP, e.g., the commonly used PAmCherry [16], can be imaged in the second phase by UV-light mediated photoactivation. The benefits of such an approach are substantial. Since both targets are imaged in the same channel, chromatic aberration is eliminated. Both FP-tags can be endogenous, requiring no additional staining, and the above chosen FPs are bright and show no oligomerization tendencies [17]. In addition, as they fluoresce with a maximum in the 570–590 nm range upon 561 nm illumination, possible auto-fluorescence of biological samples and phototoxicity are reduced when compared to techniques using shorter wavelength channels [14,18]. This makes this labeling choice favorable compared to other, e.g., non-red, less bright and photostable or potentially self-oligomerizing pcFPs and paFPs such as paGFP [9,19], psCFP2 and Dronpa [10] or PAtagRFP [20] and PAmKate [7]. Finally, our strategy works hand in hand with existing, multi-channel approaches and can be easily used to expand them.

## 2. Results and Discussion

To evaluate the viability of the proposed imaging scheme, two main concerns had to be addressed. First, does PC illumination have any effect on the paFP? Second, is it possible to sufficiently eliminate the pcFP imaged by PC to not cause any cross-talk during the second imaging phase as any residual, non-converted pcFP molecule would also be read-out by UV light. To test this, we performed experiments on fixed *Escherichia coli* cells, where the native RNA polymerase (RNAP) protein was endogenously tagged with either Dendra2 or PAmCherry (Supplementary Material and Methods). RNAP molecules appear in large nucleoid-like patterns as they decorate the bacterial chromosome. Their numbers per unit of cell size are relatively stable when expressed under the native promoter, making RNAP a satisfactory target for quantitative controls [21].

First, we determined the minimum dose of 488 nm light required to permanently bleach all Dendra2-RNAP molecules in the *E. coli* cells (Figure S1a). A bleaching approach like this would be necessary in a worst-case scenario, where full readout of the PC-suitable pcFP is not feasible, e.g., in cases where the POI is highly abundant. We then evaluated the effect of this high 488 nm dose on the PAmCherry molecules (Figure S1a). While the vast majority of the Dendra2-RNAP read-out in the red channel was gone after 30 s of illumination with 1 kW/cm^2^ of 488 nm light, we used 120 s for subsequent imaging since that is where the bleaching curve stabilized. Using the same settings, we found that 488 nm illumination slightly increased the fluorescence intensity in cells with PAmCherry-tagged RNAP, suggesting some minor activation (Figure S1a). With this information we were able to design a worst-case scenario control imaging scheme (Figure S1b, left). To quantify the loss of PAmCherry during the first, PC-photoconversion stage of imaging and potential bleed-through of Dendra2 into the second UV-photoactivation phase, we performed localization counting on *E. coli* cells expressing RNAP-Dendra2 and RNAP-PAmCherry under several different illumination schemes (Figure S1b, right). RNAP-Dendra2 produced a mean of 1092 ± 175 (standard deviation) localization counts/μm^2^ converted by primed conversion (I) and a mean of 999 ± 131 localization counts/μm^2^ (II) by UV photoconversion which is in agreement with previous results on RNAP numbers [21,22] and reconfirms data of a recent study which did not show any differences in quality and quantitative numbers obtained in sptPALM tracking studies comparing UV and PC photoconversion of Dendra2 [14]. After 120 s of bleaching with 1 kW/cm^2^ 488 nm light, Dendra2 was activated by UV conversion and produced 54 ± 18 localization counts/μm^2^ (III), which is at the level of false positive localization counts in wild type *E. coli* (data not shown), confirming that little to no Dendra2 survives the bleaching step. Next, we evaluated the UV readout of RNAP-PAmCherry, resulting in 1054 ± 217 counts/μm^2^ (IV), which is comparable to the Dendra2 PC readout. To test the effects of high intensity 488 nm light on PAmCherry, RNAP-PAmCherry expressing cells were illuminated with the same high intensity of 1 kW/cm^2^ 488 nm light for 120 s, and then read out with UV activation, producing 916 ± 182 counts/μm^2^ (V), further suggesting that 488 nm light activates PAmCherry with low efficiency. This, however, proved not to be an issue during the conversion imaging phase, where the overall dosage of 488 nm light is much lower (less than 0.1% of the bleaching dose of 1 kW/cm^2^ used in this experiment). PAmCherry read out by PC activation, produced 45 ± 19 counts/μm^2^ (VI), again at the level of background in wild type *E. coli*. To eliminate cross-talk, we thus implemented a brief, ~10 s pre-bleaching step with the 561 nm light readout laser before reading out the PAmCherry, bleaching away any pre-activated FP. These findings suggest that if the FP in the first phase of imaging is read out in full and a high intensity 488 nm post-bleaching step is not required, loss of PAmCherry is completely negligible. In such a case, both phases allow for a full quantitative SMLM readout.

**Figure 1:**
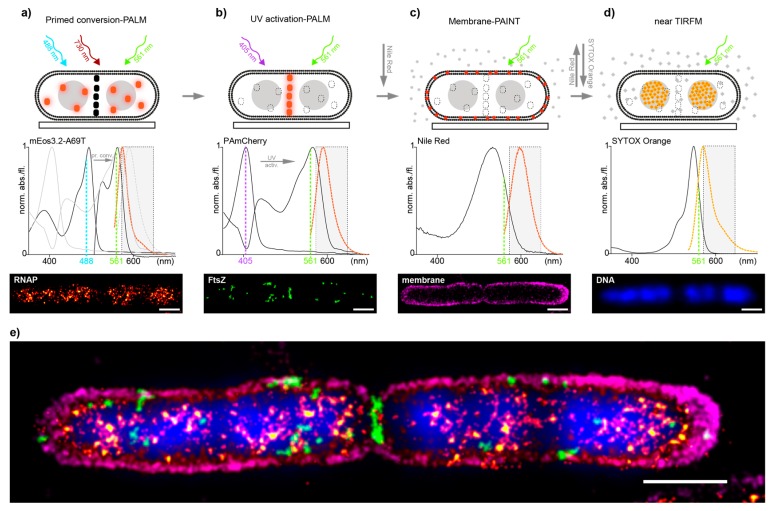
(**a**–**d**) Multi-color imaging workflow in *E. coli* cells. Top: scheme representing the acquisition sequence by first imaging RNAP-mEos3.2-A69T (orange dots) by primed conversion-PALM, not activated FtsZ-PAmCherry molecules are depicted as black dots (**a**); FtsZ-PAmCherry (orange dots) by UV activation-PALM, permanently bleached RNAP-mEos3.2-A69T are presented as dashed-lined white dots (**b**); The outer membrane by PAINT using NileRed- red dots depict fluorescent fraction of NileRed molecules in the membrane, whereas gray dots represent non-fluorescent NileRed in buffer/medium (**c**) and last the DNA stained by SYTOX Orange (orange diamonds) and read-out by near TIRF microscopy (**d**). Middle: Measured absorption and emission spectra of all four fluorophores used (Supplementary Material and Methods). Dashed line rectangle represents the optical bandpass-filter used to visualize the fluorescence channel of all four acquisitions. Bottom: SMLM image reconstructions (**a**–**c**) and nearTIRF-snapshot (**d**) recorded; (**e**) Overlay of all individual images to show the mutual organization of imaged compounds, aligned by fiducial markers (Supplementary Material and Methods). Localization precision of the channels after drift correction (whole ROI, NeNA values): mEos3.2-A69T (RNAP) 12.1 nm, PamCherry (FtsZ) 12.3 nm, NileRed (membrane) 11.8 nm, scale bars: 1 μm.

Encouraged by the promising results of the controls, we decided to evaluate how well our approach performs in dual-color imaging of different biomolecules in *E. coli* and in a second step, how well it integrates with additional correlative imaging schemes such as membrane point accumulation for imaging of nanoscale topography (PAINT) and DNA stains [23]. To test this, we utilized the imaging scheme on an *E. coli* strain where RNAP was endogenously tagged with the PC-suitable mEos3.2-A69T mutant and transformed with the pJB063 plasmid bearing the sequence for FtsZ-PAmCherry. The green-to-red pcFP mEos3.2-A69T mutant has proven, like Dendra2, to be an excellent candidate for tagging native proteins in our previous work but with a 15–20% higher photoncount for single-molecule-spots than Dendra2—thus allowing for more precise sptPALM data [14]. RNAP-mEos3.2-A69T was imaged first and read out in full (Figure 1a), followed by a full readout of FtsZ-PAmCherry (Figure 1b). Nile-red was then added to the imaging solution to image the membrane by PAINT [24] (Figure 1c) and was finally replaced with a Sytox orange containing solution, to stain the DNA (Figure 1d), for a total of four effective colors all in the orange-red channel (Figure 1e).

**Figure 2:**
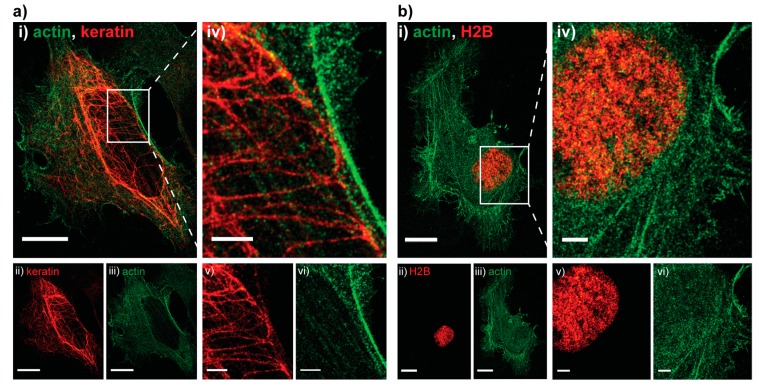
Two examples of Hela cells imaged with sequential PC activation. (**a**): (**i**) Dual-color PALM image of actin tagged with PamCherry (green) and keratin tagged with Dendra2 (red); (**ii**,**iii**) split channel PALM images of the same cell. Localization precision of the channels after drift correction (whole ROI, NeNA values): Dendra2 (red, keratin) 17.2 nm, PamCherry (green, actin) 17.4 nm, scale bars 5 μm; (**iv**–**vi**) zoom in on an area with details only visible in the super resolved image, scale bars 2 μm; (**b**): (**i**) Dual-color PALM image of actin tagged with PAmCherry (green) and H2B tagged with Dendra2 (red) and split channel images of the same cell (**ii**,**iii**). Localization precision of the channels after drift correction (whole ROI, NeNA values): Dendra2 (red, H2B) 16.1 nm, PamCherry (green, actin) 18.9 nm, scale bars 5 μm; (**iv**–**vi**) zoom in on the nucleus, showcasing the absence of bleed through (dark background on (**vi**)), scale bars 2 μm.

Next, we assessed how well the technique performs on larger samples in other organisms by imaging HeLa cells transiently transfected with a combination of an actin-PAmCherry plasmid together with either a keratin-Dendra2 plasmid (Figure 2a) or an H2B-Dendra2 plasmid (Figure 2b), details can be found in Supplementary Material and Methods. As before, the pcFP, Dendra2, was read out first. Due to the large size of the cell and large number of (overexpressed) molecules a full read-out was rather time consuming, thus we decided to bleach the residual Dendra2 molecules after obtaining a sufficient number of localizations for image reconstruction. The intensity of 488 nm bleaching step had to be increased to 4 kW/cm^2^ in order to fully bleach the residual Dendra2 molecules above and below the imaging plane. No visible cross talk was present on any of the images, as showcased by the dark background around the H2B (Figure 2b(v)).

A major advantage of the approach is its potential for use in living cells, especially for targets which are difficult-to-reach by extrinsic fluorescent markers or have precluded SMLM read-out channels due to low signal-to-noise/high background, e.g., arising from intracellular autofluorescence. Like all (sequential) SMLM techniques, our approach is limited by the temporal resolution needed for structural studies of abundant proteins but offers the read-out of two different proteins by intrinsic FP labels with excellent SMLM properties to measure their molecule dynamics within the same compartment or cell [25].

We thus first tested our *E. coli* strain under live conditions (Figure S2a), measuring diffusion dynamics for RNAP and FtsZ (Figure S2b,c) as reported before [14,26] and could confirm the cell survival after sptPALM imaging [14] (Figure S2d).

We then challenged our approach by creating a strain of the fission yeast *Schizosaccharomyces pombe*, where the DNA binding protein cbp1 and the centromeric histone variant protein cnp1 were endogenously tagged with Dendra2 and PAmCherry, respectively (Supplementary Material and Methods). Introducing fluorophores for live cell SMLM imaging into *S. pombe* is difficult. In our hands, only few extrinsic labels can be delivered to protein-tag-fusions but are prone to unspecific background staining due to the cell wall and dense intracellular environment and do not blink in the intracellular living yeast environment (unpublished data). Additionally, dual-color SMLM imaging including a green FP does not provide satisfactory single molecule signal above the intracellular background (unpublished data). Thus, even most recent SMLM studies of living *S. pombe* have remained exclusively single-color using mEos2/mEos3 [27,28] which is in contrast to mammalian cell studies, where multi-color SMLM introducing dye molecules that blink in the intracellular environment has been applied in various studies [1,3].

**Figure 3:**
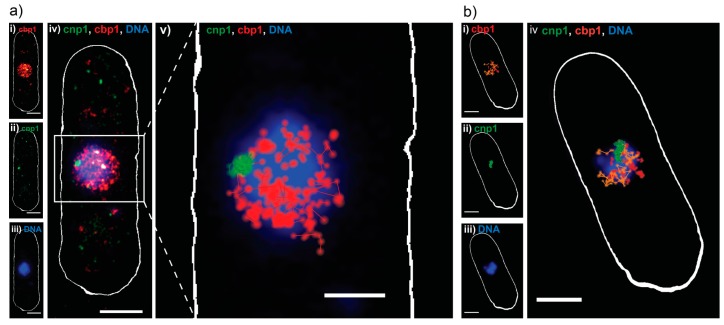
Sequential dual-color PALM on *S. pombe*. (**a**) Dual-color PALM image of a representative *S. pombe* cell, expressing the DNA binding protein cbp1 tagged with Dendra2 (red) and the centromeric protein cnp1 tagged with PAmCherry (green) and a widefield fluorescence image of DAPI stained DNA (blue): (**i**–**iii**) Split channels of the same cell and (**iv**) combined; (**v**) single particle track analysis performed on the same data, with tracks ≥3 consecutive frames represented in red (cbp1) and green (cnp1), on top of DNA (blue). Scale bars 2 μm; (**b**) single particle tracking analysis done on a different *S. pombe* cell: (**i**) single particle tracks of cbp1 ≥4 consecutive frames, color coded for their apparent diffusion coefficient D as calculated from a MSD analysis of the individual tracks, orange for fast moving particles (D > 0.26 μm^2^/s) and red for slow or immobile (D < 0.26 μm^2^/s); (**ii**) single particle tracks of cnp1 ≥ 4 consecutive frames; (**iii**) widefield fluorescence image of DNA in the same cell, after fixation; (**iv**) overlay of all three channels. Scale bars 2 μm.

As in *E. coli*, control measurements characterizing the viability of our living cells revealed no negative effect of our imaging approach on the cellular growth (Figure S3a–d). As before, Dendra2 was read out first, with lower 561 nm readout laser intensities and shorter exposure times, to capture the movement of single molecules (Figure 3a,b(i)). PAmCherry-cnp1 was successfully read out in the second phase, with no apparent crosstalk (Figure 3a,b(ii)). The cells were then fixed and stained with DAPI (Figure 3a,b(iii)), since Sytox Orange penetrated the thick yeast cell wall poorly in our hands (data not shown). As expected, reconstructed PALM images showed cbp1 is present throughout the nucleus and co-localizes with DNA, while cnp1 appears as a single centromeric spot at the edge of the DNA (Figure 3a,b(iv)). We then applied a custom single particle tracking algorithm on the same data and visualized the results (details in materials and methods), which showed that the cbp1 diffuses around the nucleus, while the cnp1 is largely immobile (Figure 3a(v)). Finally, we calculated diffusion coefficients for tracks longer than four consecutive frames, revealing a mobile (fast) and an immobile (slow) fraction of cbp1 (Figure 3b(iv)) and exemplary mean squared displacement (MSD)-curves in Figure S4a). Imaging fixed cbp1 molecules as a control under otherwise same conditions reveals that the slow fraction is indeed not moving within the precision of the sptPALM measurements and represents immobile, DNA-bound cbp1 molecules whereas the fraction of mobile cbp1 with a more heterogeneous distribution of diffusion coefficients represents DNA-associated, but mobile cbp1 molecules (Figure S4b(i,ii)) [29,30].

## 3. Materials and Methods

Strain constructions for *E. coli* and *S. pombe* cell lines, transient transformations for mammalian samples, protein purification, live and fixed cell sample preparations for *E. coli*, *S. pombe* and mammalian cells as well as the microscopic and spectroscopic equipment are described in detail in the Supplementary Information (Supplementary Material and Methods).

### 3.1. Imaging Procedures

#### 3.1.1. Influence of High Intensity of 488 nm Light on PAmCherry and Dendra2

To determine, whether high intensity of 488 nm light leads to irreversible bleaching of Dendra2 molecules while leaving PAmCherry intact, we imaged fixed MG1655 rpoC_Dendra2 and MG1655 rpoC_PAmCherry cells. First, cells were illuminated with 1 kW/cm^2^ of 488 nm for 0 to 180 s (Figure S1a) and then illuminated for 15 s with 2.5 W/cm^2^ of 405 nm light to perform photoactivation or photoconversion of non-bleached fluorescent proteins. In last step, three 60 ms frame snapshots with 800 W/cm^2^ of the 561 nm laser were taken.

#### 3.1.2. Quantitative Controls-RNAP Molecule Counting

To evaluate possible false positives of residual Dendra2 and the amount of pre-activated PAmCherry of the second phase of imaging (Dendra2 converted via UV light or PAmCherry preactivated via 488 nm light), a set of control experiments was performed (Figure S1b). In experiment (I) fixed MG1655 RNAP-Dendra2 cells were illuminated with three lasers: 488 nm pulsed every 20th frame and continuous 561 and 730 nm. Laser intensities were: 100–2500 mW/cm^2^ of 488 nm (intensity was gradually increased during movie acquisition to keep the number of detected spots roughly on the same level), 800 W/cm^2^ of 561 nm and 450 W/cm^2^ of 730 nm. Movies were recorded at 16.67 Hz image acquisition (60 ms per frame) until no new fluorescent spots appeared to ensure for a full read-out of the FP. In experiment (II), MG1655 RNAP-Dendra2 was illuminated by conventional UV-conversion PALM: The 405 nm laser was pulsed every 20th frame, gradually increasing the intensity from 250–6500 mW/cm^2^ and continuous 561 nm laser at a constant intensity of 800 W/cm^2^. The field of view (FOV) was imaged until no new fluorescent spots appeared anymore. As a negative control (III), MG1655 RNAP-Dendra2 was first illuminated for two minutes with 1 kW/cm^2^ of 488 nm laser to irreversibly bleach the Dendra2 molecules. Next, the same FOV was imaged with standard UV-conversion PALM. Laser intensities were the same as for (II). The FOV was imaged for 5 min with 60 ms exposure time per frame. Experiments (IV)–(VI) were performed on the MG1655 RNAP-PAmCherry strain. Experiment (IV) was a positive control where cells were imaged with UV-conversion PALM: 405 and 561 nm laser settings were the same as for experiment (II) and the FOV was imaged until no new spots appeared. In experiment (V) MG1655 RNAP-PAmCherry cells were imaged in a similar way as cells in experiment (III) with an extended imaging time until no new spots appeared, achieving full read-out. In experiment (VI) which serves as negative control, cells were imaged as the MG1655 RNAP-Dendra2 cells in experiment (I), to evaluate the activation of PAmCherry via primed conversion. FOV was imaged for 5 min. For each control experiment two FOVs were imaged.

#### 3.1.3. *E. coli* Multi-Color Imaging

Detection of RNA polymerase (RNAP) fused with mEos3.2-A69T, FtsZ fused with PAmCherry, bacterial membrane and chromosomal DNA in fixed MG1655 rpoC_mEos3.2-A69T+pJB063 cells was performed sequentially (Figure 1). First, RNAP-mEos3.2-A69T molecules were imaged by primed conversion [14] (Figure 1a). Briefly, the sample was illuminated with three lasers: 488 nm pulsed every 20th frame and continuous 561 and 730 nm light. Laser intensities were: 100–2500 mW/cm^2^ of 488 nm (intensity was gradually increased during movie acquisition), 800 W/cm^2^ of 561 nm and 450 W/cm^2^ of 730 nm. Movies were recorded at 16.67 Hz image acquisition (60 ms per frame) until no new spots appeared. After primed conversion PALM, the sample was illuminated for one minute with 1 kW/cm^2^ of 488 nm laser to irreversibly bleach residual green form mEos3.2-A69T. In the second phase (Figure 1b) FtsZ-PAmCherry molecules were detected with UV-activation PALM: 405 nm laser pulsed every 20th frame (intensity gradually increasing from 250–6500 mW/cm^2^) and continuous 800 W/cm^2^ 561 nm laser at 60 ms until all PAmCherry was read out. After finishing imaging both, RNAP-mEos3.2-A69T and FtsZ-PAmCherry, Nile Red (Sigma-Aldrich, Darmstadt, Germany) was added (final concentration in imaging buffer: 7 nM) to visualize the bacterial membrane in the same cells by PAINT microscopy [24] (Figure 1c). Movies were recorded for 7 min with 60 ms exposure time per frame and the sample was illuminated with constant 1.2 kW/cm^2^ of 561 nm laser. In the last, fourth step (Figure 1d), chromosomal DNA was visualized by addition of SYTOX Orange (Thermo Fischer, Darmstadt, Germany) to a final concentration 20 nM. One-frame 60 ms snapshots were taken with low intensity 561 nm laser (10 W/cm^2^). For all fixed bacteria images, the Nearest Neighbor Analysis (NeNA) values [31] were in the range of 10–15 nm, exact values of the exemplary images are given in the respective captions.

Single-particle tracking PALM (sptPALM) imaging (Figure S2) of rpoC_mEos3.2-A69T+pJB063 cells was performed on a heating stage and heated objective at 32 °C. As for the fixed samples, RNAP-mEos3.2-A69T was imaged first and FtsZ-PAmCherry was imaged afterwards. Applied laser intensities were: 2 W/cm^2^ of 405 nm, 600 mW/cm^2^ of 488 nm, 450 W/cm^2^ of 730 nm and 800 W/cm^2^ of 561 nm laser light. 405 and 488 nm lasers were pulsed every 20th frame. Movies were recorded at 77 Hz image acquisition rate for two minutes to follow the diffusing RNA polymerase and at 33 Hz image acquisition rate for five minutes to follow the FtsZ protein. For the FtsZ imaging we used slower acquisition mode to exclude cytosolic FtsZ molecules—as they diffuse much faster than FtsZ built into the ring structures, their point-spread functions are blurred, thus most of the cytosolic FtsZ molecules are not included in further analysis steps. This approach was first used by Etheridge et al. in their work [32]. mEos3.2-A69T molecules were converted by primed conversion, while PAmCherry was photoactivated using UV. Right after recording the RNAP-mEos3.2-A69T molecules, a two minute bleaching step to bleach the remaining unconverted mEos3.2-A69T molecules was performed using 1 kW/cm^2^ 488 nm laser light illumination to avoid false positives during the FtsZ-PAmCherry readout. After sptPALM imaging, the cells of the imaged FOVs were tested for their cell viability. For this, all FOVs were imaged under bright light for three hours with two minutes interval to quantify their cellular growth after the sptPALM experiment.

#### 3.1.4. HeLa Dual-Color Imaging

Keratin-Dendra2 or H2B-Dendra2 were imaged first, activated with 450 W/cm^2^ of the 730 nm laser and 100–2500 mW/cm^2^ of the 488 nm laser (pulsed every 20th frame) with the intensity gradually increased to keep the number of localizations per frame constant and read out with the 561 (800 W/cm^2^) laser for 20 thousand frames at an exposure of 60 ms per frame. The sample was then illuminated with 4 kW/cm^2^ of the 488 nm laser for 2 min, to bleach any residual Dendra2. Actin-PAmCherry was imaged second, activated with the 405 nm laser, pulsed on every 12th frame, with the intensity gradually adjusted from 250–6500 mW/cm^2^ and read out with constant 561 illumination at 800 W/cm^2^ for 10 to 20 thousand frames at an exposure time of 60 ms per frame. Lasers were angled to achieve near-TIRF illumination. For all mammalian images, the NeNA values were in the range of 15–20 nm, exact values of the exemplary images are given in the respective captions.

#### 3.1.5. *S. pombe* Multi-Color Imaging

Cbp1-Dendra2 was imaged first, activated with 450 W/cm^2^ of the 730 nm laser and 100–2500 mW/cm^2^ of the 488 nm laser (pulsed every 20th frame) with the intensity gradually increased to keep the number of localizations per frame constant and read out with the 561 (500 W/cm^2^) laser for 20 thousand frames at an exposure of 20 ms per frame. The sample was then illuminated with 2 kW/cm^2^ of the 488 nm laser for 1 min, to bleach any residual Dendra2. Cnp1-PAmCherry was imaged second, activated with the 405 laser, pulsed on every 12th frame, with the intensity gradually adjusted from 250–6500 mW/cm^2^ and read out with constant 561 illumination at 500 W/cm^2^ for 10 thousand frames at an exposure time of 20 ms per frame. Finally, cells were fixed with 1% paraformaldehyde (Sigma-Aldrich, F8775) without moving the sample and stained for DNA with 1 μg/mL DAPI, read out by the 405 nm laser (10 W/cm^2^).

#### 3.1.6. Viability Controls

*S. pombe* cbp1-Dendra2 cnp1-PAmCherry cells were grown in YES medium at 25 °C overnight, then inoculated into fresh YES to a starting OD_600_ of 0.1 and grown for ~6 h before imaging. 1 mL of culture was spun down at 500× *g* for 5 min and resuspended in 10 μL fresh YES medium. Cells were immobilized on a 1% low-gelling temperature agarose pad (Sigma-Aldrich, A9414) in YES medium and covered with a coverslip previously cleaned with Hellmanex. Cells were imaged on a custom-built heating stage at 30 °C. To compare growth, 8 FOVs at least 1000 μm apart were chosen and bright field images were taken at time t_0_. The first two FOVs were imaged with PC activation 450 W/cm^2^ of the 730 nm laser and 100–2500 mW/cm^2^ of the 488 nm laser (pulsed every 20th frame) with the intensity gradually increased to keep the number of localizations per frame constant and read out with the 561 (500 W/cm^2^) laser for 20 thousand frames at an exposure of 15 ms per frame. The next two FOVs were imaged with the same conditions but also post-bleached with 2 kW/cm^2^ of the 488 nm laser for 2 min. Two further FOVs were then imaged and post-bleached as described above, then also imaged with UV activation, 405 laser pulsed on every 12th frame, with the intensity gradually adjusted from 250–6500 mW/cm^2^ and read out with constant 561 illumination at 500 W/cm^2^ for 5 thousand frames at an exposure time of 30 ms per frame. A bright field of each position was taken every 10 min for 12 h after imaging with an automated μManager script, though FOVs were lost after ~4 h due to drift. Exemplary cells are depicted in Figure S3.

### 3.2. Data Analysis

#### 3.2.1. Analysis of the Influence of High Intensity 488 nm Images

Acquired three-frame snapshots were analyzed with a custom written script in *Fiji software (ImageJ 1.51f*) [33]. Cell FOVs were extracted by identifying individual bacterial shapes from fluorescence averages excluding overlapping or out-of-focus cells. Averaged fluorescence intensity for each cell was used as a parameter of bleaching degree (Figure S1b).

#### 3.2.2. Super-Resolution Image Reconstruction of Bacterial and Mammalian Multi-Color Images

Localizations of the fitted single fluorescent spots were obtained by the open source software *rapidSTORM* (version 3.3) [34]. Fitting parameters were determined individually for each kind of fluorophore (Dendra2, mEos3.2-A69T, PAmCherry and Nile Red). In the next step, localization precision for all FOVs was estimated using NeNA [31] as implemented in the open-source software *LAMA* (version 16.10) [35] on a section of the image that contained no fiducial markers. For all FOVs localization precision was determined between 10 and 15 nm for bacteria and 15 and 20 nm for mammalian cells. Localization data were filtered to connect neighboring localizations in adjacent frames, to avoid several-fold counting of molecules with fluorescence lifetimes exceeding the framerate of the movie. In the last post-processing step, all localization files were drift corrected with custom written *Python* algorithms that extract and correct for fluorescent bead trajectories. Additionally, for FtsZ-PAmCherry localizations, density based clustering analysis was performed with the density-based spatial clustering of applications with noise (DBSCAN) algorithm [36], as single cytosolic FtsZ-PAmCherry proteins were abundantly present in cell. Only clustered molecules (DBSCAN parameters: *ε* = 35 nm; MinPts = 10) were used for image reconstruction of FtsZ-PAmCherry. Super-resolution image reconstructions and DNA-Sytox Orange image were stacked and superimposed in *Fiji* software. For HeLa cells, images were reconstructed with a pixel size of 20 nm.

#### 3.2.3. Single Particle Tracking in *E. coli* and *S. pombe*

Single molecule localizations were extracted from the movies with the open-source software *rapidSTORM*. Final images were reconstructed with a pixel size of 10 nm. Single particles were tracked with the help of customized tracking software written in C++ and visualized by customized software written in C++, to filter and group single molecule localizations or trajectories by their apparent diffusion coefficient (as calculated by MSD analysis). For MSD analysis, only trajectories with 4 and more steps were used for calculating the MSD values. Independent of the total length of each trajectory, only the first steps of each trajectory were used for the MSD calculation (for the first 3 Δt values) to account for heterogeneous diffusion behavior over time as well as the confinement of the yeast nucleus/*E. coli* cell. To account for the localization precision σ of the data, the constant offset of 4*σ^2^ was included into the fit-function. For diffusion coefficient statistics, only MSD fits with an R^2^ value of 0.85 or higher were used.

## 4. Summary and Conclusions

In summary, our new technique produced high quality PALM images on all tested samples. The controls showed there is little to no crosstalk between the two proteins and loss of PAmCherry is minimal. This can be further reduced by avoiding the post-bleaching step with high intensity of 488 nm light, by reading out the entire signal in the first phase of imaging and, for fixed cells, by increasing the pH to 8–8.5 where PC is most efficient [14]—making both phases of imaging fully quantitative. We have also shown that the approach is easily combined with other sequential imaging techniques, such as membrane PAINT. This correlative approach could be extended further, by e.g., substituting the Sytox Orange DNA stain for the transiently binding Hoechst-JF646 probe for DNA-PAINT [37].

Perhaps most importantly, the combination of a PC-suitable pcFP and an only UV-activatable paFP allows for dual-color SMLM imaging in living specimens, without the need for any additional staining steps, as shown in *E. coli* and *S. pombe*. Previously, this could only be done through the use of proteins with different emission spectra, such as paGFP or psCFP2. This requires imaging in different readout channels, introducing chromatic aberration, which makes overlaying the channels more difficult. Additionally, many biological samples, e.g., including *S. pombe*, can exhibit high degrees of autofluorescence in the GFP channel. Combined with the lower brightness of paGFP and psCFP2 compared to red paFPs, this results in a limited achievable resolution. Also, the superior brightness of FPs such as Dendra2, mEos3.2-A69T and PAmCherry allows for single particle tracking experiments, where brightness is essential due to shorter exposure times and lower excitation light intensities. That being said, our approach could potentially be combined with other FPs that emit in different channels, further expanding the palette of available colors for live-cell imaging or be used in tandem with enzyme tags such as Halo-tag [38] and SNAP-tag [39] together with membrane-permeable dyes (and e.g., as well using sequential orange stainings like TMR and paJF549).

In conclusion, our measurements demonstrate that this new method can be applied to various targets in different organisms and can be advantageously combined with existing imaging schemes. As it is an aberration-free, live-cell compatible method, which is simple to implement on conventional SMLM systems (even when using a red instead of an infrared laser source, PC can be efficient enough for SMLM imaging [13,14]), we believe it is a valuable addition to the current SMLM toolbox.

## Supplementary Materials

Supplementary materials can be found at www.mdpi.com/1422-0067/18/7/1524/s1.

## Abbreviations

FP	Fluorescent protein
paFP	Photoactivatable fluorescent protein
PAINT	Points accumulation for imaging in nanoscale topography
PALM	Photoactivated localization microscopy
PC	Primed conversion
pcFP	Photoconvertible fluorescent protein
SMLM	Single molecule localization microscopy
