# Supplementary material for: Combining Primed Photoconversion and UV-Photoactivation for Aberration-Free, Live-Cell Compliant Multi-Color Single-Molecule Localization Microscopy Imaging
Source: Int J Mol Sci. 2017 Jul 14;18(7):1524. doi: 10.3390/ijms18071524 (PMC5536014; doi:10.3390/ijms18071524)
Supplement: Supplementary file 1 [file ijms-18-01524-s001.zip › ijms-205949-supplementary.pdf]

Supplementary

Combining Primed Photoconversion and UV-Photoactivation for Aberration-Free, Live-Cell Compliant Multi-Color Single-Molecule Localization Microscopy Imaging

David Virant, Bartosz Turkowyd, Alexander Balinovic and Ulrike Endesfelder *

Department of Systems and Synthetic Microbiology, Max Planck Institute for Terrestrial Microbiology & LOEWE Center for Synthetic Microbiology (SYNMIKRO), Karl-von-Frisch-Str. 16, 35043 Marburg, Germany

* Correspondence: ulrike.endesfelder@synmikro.mpi-marburg.mpg.de; Tel.: +49-6421-28-21619

Figure S1 –S4

Table S1

Supplementary Materials and Methods

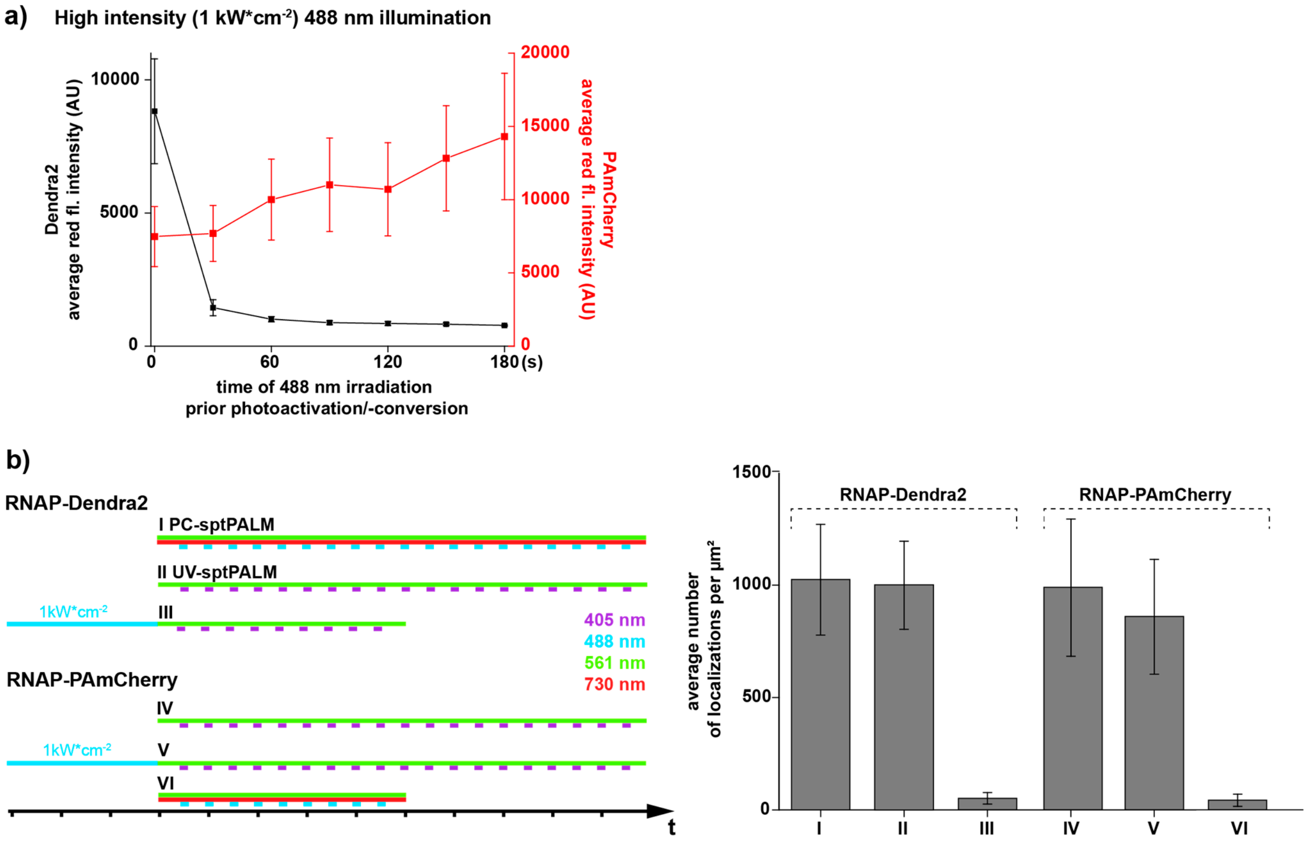

Figure S1: (a) Influence of high intensity (1 kW/cm²) 488 nm light illumination measured by the average red fluorescence intensity of PAmCherry and Dendra2 as a function of time of exposure by 488 nm light prior photoactivation/-conversion (for details, see materials and methods). Black graph: Dendra2 average fluorescence intensity; red graph: PAmCherry average fluorescence intensity. **(b) Left:** schemes representing illumination sequences for MG1655 RNAP-Dendra2 and MG1655 RNAP-PAmCherry samples serving as quantitative controls. Violet line: 405 nm light, blue line: 488 nm light, green line: 561 nm light, red line: 730 nm light. More details and laser intensities can be found in the materials and methods section. **Right:** average number of RNAP localizations per μm² identified during the imaging. **(I)** MG1655 RNAP-Dendra2 cells imaged with primed conversion-PALM. *n* = 13 cells; **(II)** MG1655 RNAP-Dendra2 cells imaged with UV-PALM. *n* = 21 cells; **(III)** MG1655 RNAP-Dendra2 cells illuminated with 488 nm light before imaging and imaged with UV-PALM. *n* = 21 cells; **(IV)** MG1655 RNAP-PAmCherry cells imaged with UV-PALM. *n* = 16 cells; **(V)** MG1655 RNAP-PAmCherry cells illuminated with 488 nm light before imaging and imaged with UV-PALM. *n* = 13 cells; **(VI)** MG1655 RNAP-PAmCherry cells imaged with primed conversion-PALM. *n* = 21 cells.

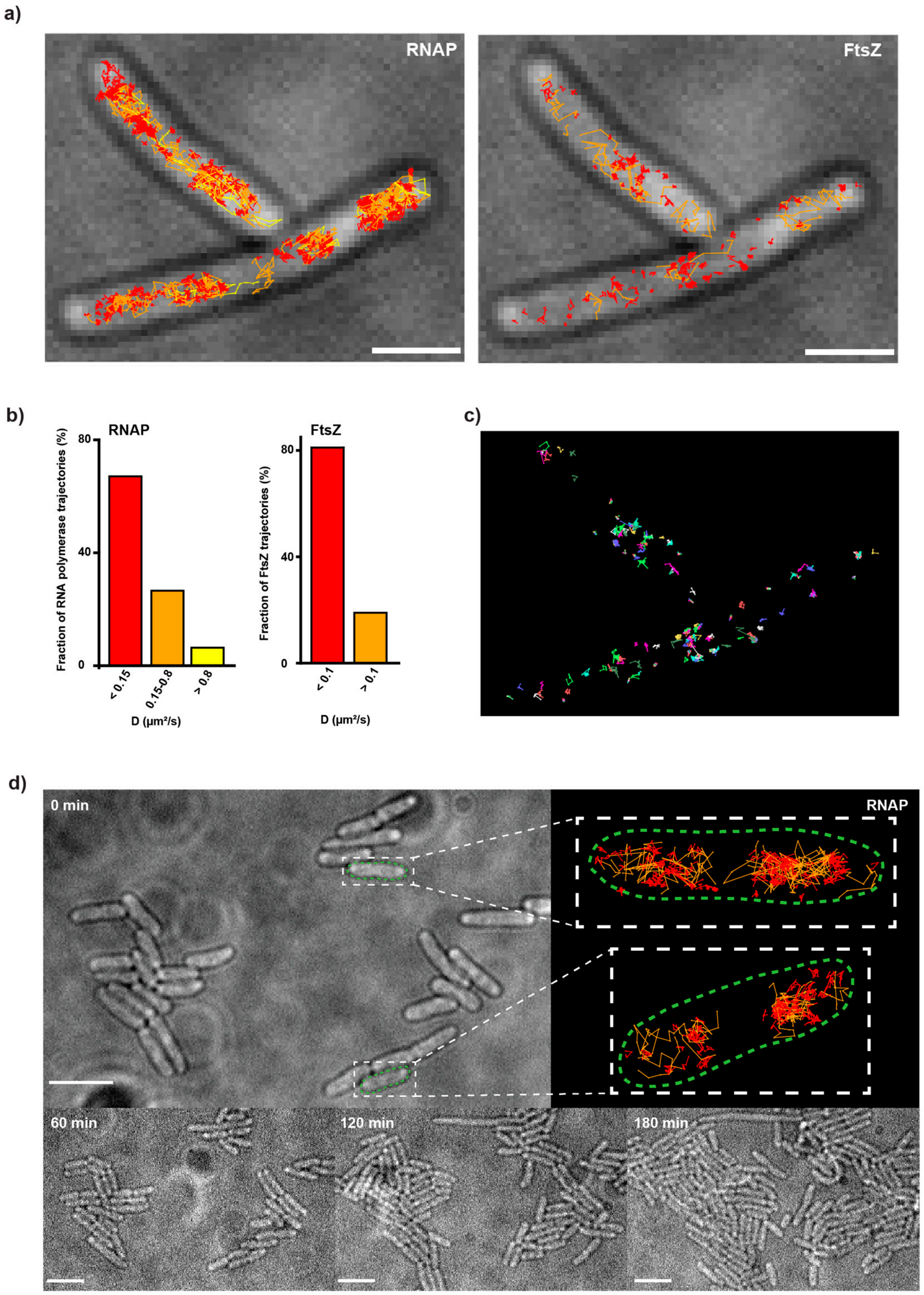

Figure S2: Live-cell single-particle tracking PALM (sptPALM) in *E. coli* cells. (a) Trajectories of RNAP molecules (left) and FtsZ (right). The individual trajectories are color coded by their apparent diffusion coefficient D and classified into categories. RNAP: 0.0–0.15 μm²/s (red), 0.15–0.8 μm²/s (orange), faster than 0.8 μm²/s (yellow) and FtsZ: 0.0–0.1 μm²/s (red), faster than 0.1 μm²/s (orange). This classification is based on recent literature on the protein dynamics [1,2] and chosen to be the same as in the histograms in **(b)**. Scale bar: 2 μm. **(b)** RNAP and FtsZ diffusion coefficient distribution histograms. RNAP molecules were classified by their diffusion coefficient as follows: slower than 0.15 μm²/s (67% of the whole population), faster than 0.15 and slower than 0.80 μm²/s (27%), and faster than 0.80 μm²/s (6%). $n = 471$ trajectories. This is in accordance with literature values [2,3]. FtsZ molecules were sorted into two classes: slower than 0.1 μm²/s (81% of the population- structural FtsZ) and faster than 0.1 μm²/s (19%). These values are also in

accordance with literature values [1]. n = 174 trajectories. **(c)** FtsZ trajectories of the structural FtsZ molecules (D < 0.1 µm²/s) shown by distinct colors to visualize individual trajectories. **(d)** Cell growth after dual-color sptPALM imaging. Cells are not affected by the in total 9-minute-read-out (2 min for RNAP-mEos3.2-A69T, 2 minutes of 488 nm bleaching of unconverted mEos3.2-A69T and 5 min for FtsZ-PAmCherry (due to the slower frame rate of 30 ms to obtain the same number of images as for RNAP-mEos3.2-A69T imaged by 13 ms frames) imaging procedure. Big bright light image: cells at the beginning of the experiment. On the right, two examples of RNAP trajectories in single cells are shown; red trajectories are for slowly diffusing RNAPs with D < 0.15 µm²/s and orange trajectories for RNAP with 0.15 µm²/s < D < 0.8 µm²/s. Bottom bright light images: cells after one, two and 3 h of growth. Scale bars: 5 µm.

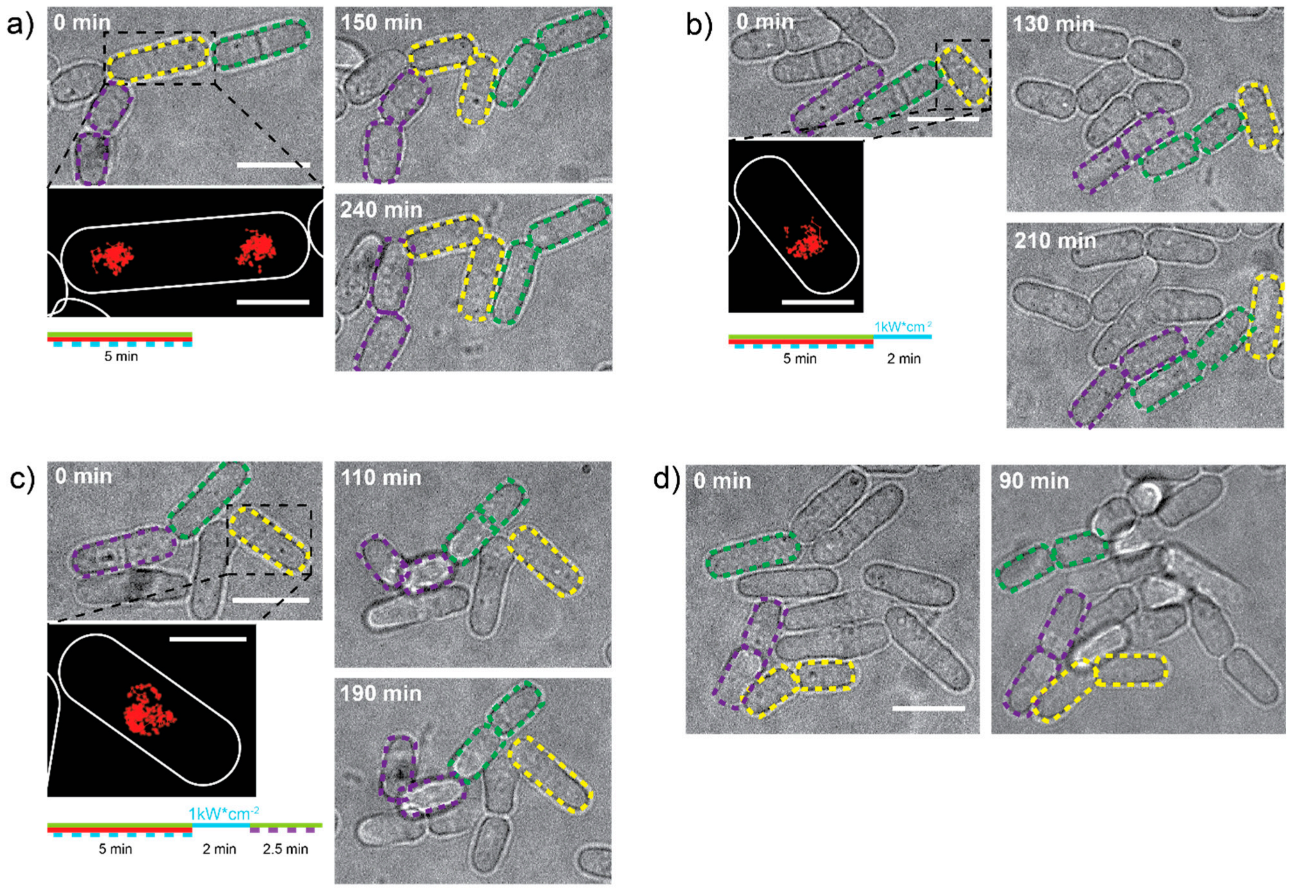

Figure S3: Comparison of *S. pombe* growth after different illumination schemes. Example cells are marked with dashed lines. Following the color-coded dashed lines, it is visible that the cells continue dividing and growing after imaging. Details experiment parameters are described in materials and methods. **(a)** Cell growth after 5 min of PC-PALM. **(b)** Cell growth after 5 min of PC-PALM and 2 min of 488 nm light post-bleaching. **(c)** Cell growth after 5 min of PC-PALM, 2 min of 488 nm light post-bleaching and 2.5 min of UV-PALM. The dose of UV light is not high enough to prevent cells from dividing/growing. **(d)** Control cells which were not illuminated by any laser light. Scale bars bright-field images 10 µm. Insets display examples of single-particle-trajectory data with a length >4 frames, obtained for cbp1-Dendra2, scale bars insets 4 µm. Colored lines beneath spt-PALM images represent the illumination scheme used in the experiment as described in **Figure S1**.

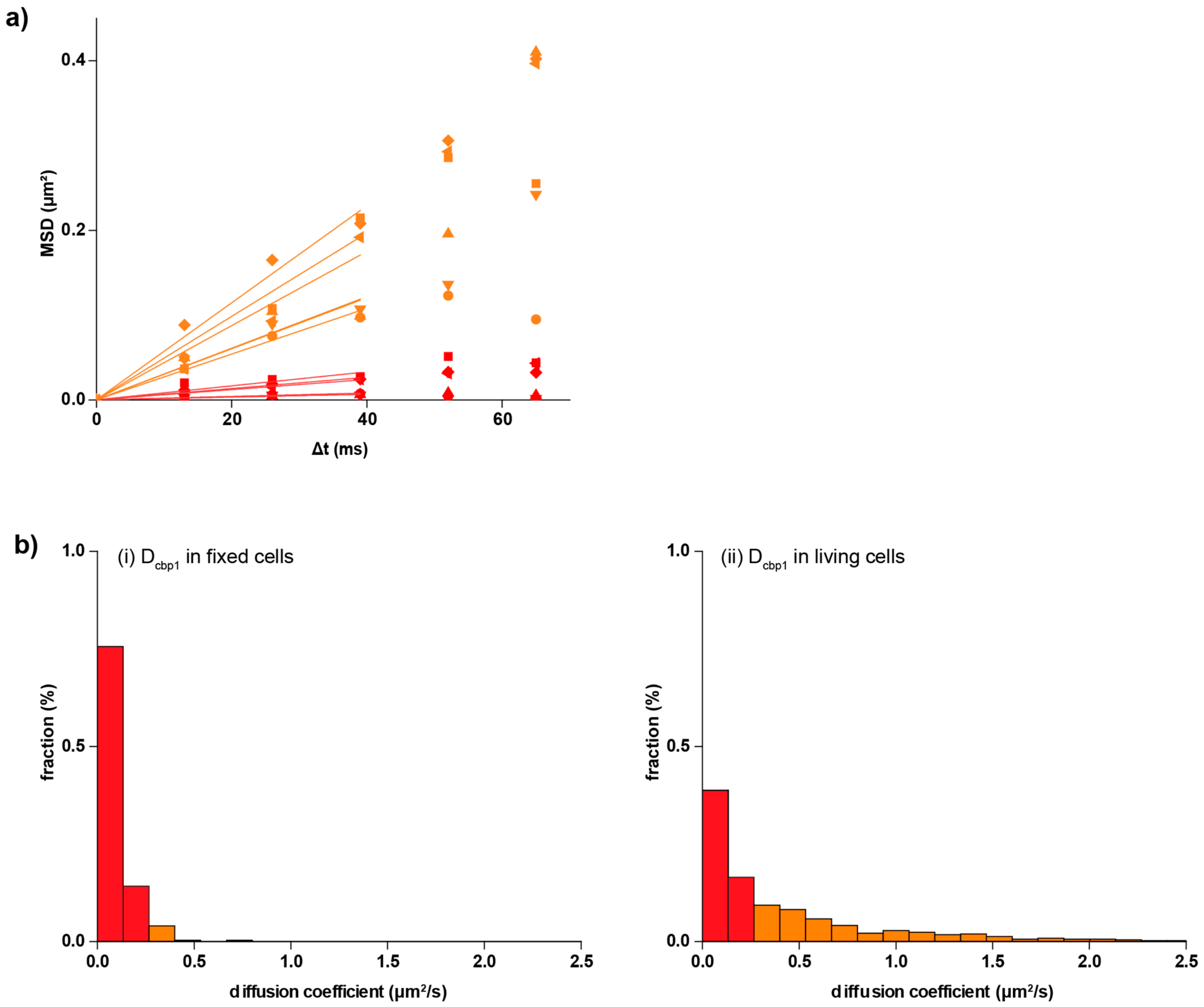

Figure S4: (a) Example fits of calculated MSD values, color coded red for MSDs corresponding to diffusion coefficients below 0.26 µm²/s and orange for MSDs corresponding to diffusion coefficients above 0.26 µm²/s. **(b)** Histograms of cbp1 diffusion coefficients in fixed **(i)** and living **(ii)** cells. Histogram bins are color coded as trajectories in **Figure 3b**, red for diffusion coefficients lower than 0.26 µm²/s and orange for diffusion coefficients above 0.26 µm²/s. **(i)** In fixed cells, the majority of analyzed trajectories displayed diffusion coefficients lower than 0.26 (~95%). All the present cbp1 molecules are expected to be immobilized by aldehyde fixation which results in a small, non-zero diffusion coefficient in agreement with the measurement error made within the localization precision. Some free-floating background may remain, resulting in a few detected mobile molecules with diffusion coefficients above 0.26 µm²/s. **(ii)** In live cells, a significant fraction (~55%) of immobile, DNA-bound cbp1 is clearly visible, while the rest of the protein moves with varying velocity. Only trajectories with a length of >4 frames and MSD-curves fitted with an R-squared value >0.85 were used in the analysis. The bin size of 0.13 was calculated using the Freedman-Diaconis rule. Number of diffusion coefficients plotted is 275 for fixed cells and 462 for live cells.

Table S1. Strains, plasmids and construction primers used in this study

Strain	Description	Reference
Escherichia coli strains		
**MG1655 rpoC_mEos3.2-A69T**	MG1655 RNAP-β′-mEos3.2-A69T, Cm^R	This study
**MG1655 rpoC_mEos3.2-A69T+ pJB063**	MG1655 RNAP-β′-mEos3.2-A69T + pJB063-FtsZ-PAmCherry, Cm^R, $Spect^R$	This study
**MG1655 rpoC_Dendra2**	MG1655 RNAP- β′-Dendra2, Cm^R	[2]
**MG1655 rpoC_PAmCherry**	MG1655 RNAP- β′-PAmCherry, Amp^R	[4]
**MG1655 pBAD/HisB_pamcherry1**	MG1655 pBAD/HisB_PAmCherry1, Amp^R	This study
Schizosaccharomyces pombe strains		
**cbp1_Dendra2**	C-terminal genomic integration, G418 resistance	This study
**cnp1_PAmCherry**	N-terminal genomic integration	[5]
**cnp1_PAmCherry + cbp1_Dendra2**	Combination of above strains	This study
Mammalian strains		
**HeLa H2B-pDendra2(N)+ β-Actin-PAmCherry1**	Transient expression from plasmid	This study
**HeLa Dendra2-Keratin-17+ β-Actin-PAmCherry1**	Transient expression from plasmid	This study

Plasmids for transient expression			
**Plasmid**	**Expression organism**	**Description**	**Reference**
**H2B-pDendra2(N)**	mammalian	H2B-pDendra2(N), Kan^R	Addgene 75283
**Dendra2-Tubulin-6**	mammalian	Dendra2-Tubulin-6, Kan^R	Addgene 57742
**β-Actin-PAmCherry1**	mammalian	β-Actin-PAmCherry1, Kan^R, Neo^R	[6]
**Dendra2-Keratin-17**	mammalian	KRT18-Dendra2, Kan^R	Addgene 57726
**pJB063**	bacteria	pBAD-FtsZ-pamcherry1, $Spect^R$	Gift from the Xiao Lab, USA
**pBAD/HisB_PAmCherry**	bacteria	pBAD/HisB_PAmCherry1, Amp^R	Addgene 31931

Construction primers	
**Name**	**Sequence**
**rpoC_mEos3.2-A69T_F**	CCAGCCTGGCAGAACTGCTGAACGCAGGTCTGGGCGGTTCTGATAACGAGgccattaaaccggatatgaaaatcaaactgcg
**rpoC_mEos3.2-A69T_R**	CCCCCCATAAAAAAACCCGCCGAAGCGGGTTTTTACGTTATTTGCGGActagagaataggaacttcctgccactcatc
**F_KanR**	GGCGCGCCAGATCTACTT
**R_KanR**	GACAGCAGTATAGCGACCAGC
**F_Dendra2**	GCCGGAGGCAGTGGTATGAACACCCCGGGAATTAA
**R_Dendra2**	AGAAGTAGATCTGGCGCGCCTTACCACACCTGGCTGGG
**F1_cbp1**	CAGCCTTGTAGTGAGGGTGTG
**R1_cbp1**	ACCACTGCCTCCGGCGGTGCTTCTCAAACGAGAAAGATTC

**F2_cbp1**	AATGCTGGTCGCTATACTGCTGTCTGTCTGTATTCGTTGTGCATATTTGAC
**R2_cbp1**	ACGAAGCAGTTAGCAAAAGAGAAGTACA

All primers were designed in Benchling (Benchling Inc.) and synthesized by Eurofins Genomics (Germany).

Supplementary Material & Methods

Strain constructions

***Escherichia coli* MG1655 rpoC-mEos3.2-A69T transformed with pJB063.** MG1655 expressing rpoC_mEos3.2-A69T was generated by homologous recombination using a modified lambda red recombination pKD46 protocol [7]. Briefly, the PCR-amplified (primers listed in **Table S1**) mEos3.2-A69T-sequence followed by the frt flanked chloramphenicol resistance gene from the codon-optimized mEos3.2-A69T pBAD18 plasmid [8] with auxiliary homologous overhang sequences was electroporated (5 ms, 1.8 kV) into MG1655 cells. After verifying the strain by sequencing (Eurofins, Ebersberg, Germany), the pJB063 plasmid (**Table S1**) containing an FtsZ sequence followed by the PAmCherry fluorescent protein and spectinomycin resistance gene sequences was introduced to MG1655 rpoC-mEos3.2-A69T via electroporation (5 ms, 1.8 kV). Transformed cells were selected under chloramphenicol and spectinomycin.

***Schizosaccharomyces pombe* cbp1-Dendra2**. The cloning strategy for tagging the C-terminus of the cbp1 was adapted from [9]. The *Saccharomyces cerevisiae* ADH1 terminator and kanamycin resistance gene were amplified from the PAW8 plasmid [10] using primers F_KanR and R_KanR. The Dendra2 fragment with the AGGSG linker was amplified from the pRSET-Dendra2 plasmid [11] using primers F_Dendra2 and R_Dendra2. The upstream (~600 bp) and downstream (~500 bp) *S. pombe* homologies were amplified from genomic DNA with the primer pairs F1_cbp1/ R1_cbp1 and F2_cbp1/ R2_cbp1 (all primers listed in **Table S1**). DNA fragments were assembled with overlap-extension PCR [12], using melting temperatures of the overlapping regions as the annealing temperature in the PCR. All PCRs were performed with Q5 High-Fidelity DNA polymerase (New England Biolabs, Frankfurt, Germany). 10 µL of the PCR product were transformed into wild type *S. pombe* using the Frozen-EZ Yeast Transformation II Kit (Zymo Research, Irvine, CA, USA), plated onto YES agar plates and incubated overnight at 30 °C, then replica plated onto 200 µg/mL G418 (Thermo Fisher Scientific, Darmstadt, Germany) YES agar plates and incubated at 30 °C until single colonies were visible. Genomic integration was confirmed by colony PCR and DNA sequencing.

***S. pombe* cnp1-PAmCherry1/cbp1-Dendra2.** The DNA fragment used for transformation was amplified from genomic DNA of the previously described cbp1-Dendra2 strain with the primer pair F1_cbp1/R2_cbp1 (**Table S1**). The fragment, containing upstream and downstream homologies, the Dendra2 sequence and the KanR gene was then transformed into an N-terminally tagged cnp1-PAmCherry *S. pombe* strain [5] as described earlier. Integration was confirmed by colony PCR and DNA sequencing.

Sample preparations

Escherichia coli. The strain MG1655 rpoC-mEos3.2-A69T+pJB063 was taken from a −80 °C stock and was cultured overnight at 37 °C, 210 rpm, LB with 34 µg/mL chloramphenicol and 100 µg/mL spectinomycin, reinoculated into fresh LB and grown to OD 0.1. Expression of FtsZ-PAmCherry from pJB063 plasmid was induced by arabinose (0.2% *w*/*v*). After 30 min of induction, the culture was centrifuged (2000× *g*, 3 min) to exchange the medium for fresh LB without inducer and again incubated in 37 °C, 210 rpm for two hours. Fixation was performed for 15 min with 1% paraformaldehyde (Sigma Aldrich, Munich, Germany) and cells were washed twice in 100 mM PBS (pH 7.4). Samples were stored in 100 mM PBS (pH 7.4) or immediately immobilized on 8-well slides (Ibidi, Munich, Germany) previously cleaned with 2% Hellmanex III (Hellma Analytivs, Muellheim, Germany) and coated with 0.05% poly-L-lysine.

MG1655 rpoC-Dendra2 and MG1655 rpoC-PAmCherry genomic strains were grown from −80 °C stocks overnight at 37 °C, 210 rpm in LB medium with appropriate antibiotic marker (**Table S1**), reinoculated and grown to OD 0.2. Fixation and cell immobilization were performed as above.

Prior to imaging, a 250 thousand fold dilution of FluoSphere dark red Carboxylate-Modified Microspheres (ThermoFisher, Munich, Germany) were added to the samples and allowed to settle for 5 min to serve as markers for drift correction.

For live-cell single-particle tracking of the strain MG1655 rpoC_mEos3.2A69T+pJB063, cells were inoculated into TB medium with chloramphenicol and spectinomycin (final concentration 34 and 100 µg/mL, respectively) and grown till OD 0.1. Then cells were induced for 45 min with 0.4% arabinose, washed with EZRDM medium (Teknova, Hollister, California, USA) and placed on 1% low melting agarose pads with EZRDM.

Schizosaccharomyces pombe. Yeast was grown in YES medium (5 g Yeast extract, 30 g glucose, 225 mg of each l-adenine, histidine, leucine, uracil, lysine-hydrochloride in 1 L of Milli-Q water) at 30 °C overnight, then inoculated into fresh YES to a starting OD of 0.1, grown to an OD of 1 and inoculated into EMM (Formedium, Hunstanton, UK) with 225 mg of each l-adenine, histidine, leucine, uracil, lysine-hydrochloride in 1 L of Milli-Q water to a starting OD of 0.1 and grown overnight at 30 °C. On the day of imaging, cells were inoculated into fresh EMM from the overnight culture to a starting OD of 0.1, grown at 30 °C until the cultures reached OD 0.4. For fixed cell experiments, yeast was fixed by adding 37% PFA to the growth medium to a final concentration of 1%, incubated for 10 min then washed three times with PBS. Cells were immobilized on poly-L-lysine coated Ibidi 8-well glass bottom slides, previously cleaned with a 2% solution of Hellmanex III , and then imaged at 30 °C.

HeLa cells. All cell culture reagents were obtained from ThermoFisher. HeLa cells were maintained in DMEM, supplemented with 10% fetal calf serum and 1% penicillin/streptomycin. Transfection of plasmid DNA was performed with FuGENE (Promega, Mannheim, Germany) according to the manufacturer's instruction. Two plasmid combinations were used, β-Actin-PAmCherry1 with Dendra2-Keratin-17 and β-Actin-PAmCherry1 with H2B-Dendra2 (**Table S1**). For each 6-well, 1 µg of plasmid DNA was diluted in 250 µL OptiMEM following addition of 4 µL FuGENE and incubation at room temperature for 30 min. 500 ng of each plasmid (250 ng in case of H2B-Dendra2) were used for co-transfections. Diluted plasmid DNA was then added to confluent Hela cells, and the culture medium was renewed after 3 h. Cells were subcultured in 8-well glass bottom slides (Ibidi, Germany) on the subsequent day. Fixation was performed with methanol (Carl Roth, Karlsruhe, Germany) at −20 °C for 15 min. Samples were washed with 1× PBS. Prior to imaging, a 250 thousand fold dilution of FluoSphere dark red Carboxylate-Modified Microspheres were added to the well and allowed to settle for 5 min to serve as markers for drift correction.

Purification of fluorescent proteins

The pellet of a culture overexpressing PAmCherry from a pBAD plasmid (**Table S1**) was suspended in 10 mM PBS (pH 7.4) with lysozyme (0.5 mg/mL) for 2 h, homogenized by ultrasound (UP100H, Hielscher, Germany) and centrifuged for 15 min (17000×*g*, 4 °C). FPs were purified from the supernatant by Ni-NTA spin columns followed by a buffer exchange to 10 mM PBS (pH 7.4) (Nanosep columns, VWR, Germany).

Microscope

Imaging was performed on a custom built setup based on an automated Nikon Ti Eclipse microscope, equipped with appropriate dichroic and filters (ET dapi/Fitc/cy3 dichroic, ZT405/488/561rpc rejection filter, ET525/50 or ET610/75 bandpass, all AHF Analysentechnik, Germany), and a CFI Apo TIRF 100× oil objective (NA 1.49, Nikon). All lasers (405 nm OBIS, 561 nm OBIS, 730 nm OBIS, 488 nm Sapphire; all Coherent Inc., Santa Clara, California USA) except 730 nm were modulated via an acousto-optical tunable

filter (AOTF) (Gooch and Housego, Eching, Germany). Fluorescence was detected by an emCCD (iXON Ultra 888; Andor, UK). The z-focus was controlled by a commercial perfect focus system (Nikon, Duesseldorf, Germany). Acquisitions were controlled by μManager [13].

Spectroscopy

Absorption spectra and fluorescence spectra (**Fig. 1a–d**) were measured in V-750 and FP-8500 instruments (Jasco, Gross-Umstadt, Germany), respectively, using purified PAmCherry protein with a final concentration 100 μM in 100 mM PBS (pH 7.5), NileRed diluted in acetone and Sytox Orange in ddH2O with final concentrations 1 μM for both in a 50μl UV-transparent quartz cuvette, except spectra of mEos3.2-A69T, which was taken from our previous work [2].

References

1. Niu, L.; Yu, J. Investigating intracellular dynamics of ftsz cytoskeleton with photoactivation single-molecule tracking. *Biophysical journal* **2008**, *95*, 2009–2016.
2. Turkowyd, B.; Balinovic, A.; Virant, D.; Golz Carnero, H.G.; Caldana, F.; Endesfelder, M.; Bourgeois, D.; Endesfelder, U. A general mechanism of photoconversion of green-to-red fluorescent proteins based on blue and infrared light reduces phototoxicity in live-cell single-molecule imaging. *Angewandte Chemie* **2017**.
3. Stracy, M.; Lesterlin, C.; Garza de Leon, F.; Uphoff, S.; Zawadzki, P.; Kapanidis, A.N. Live-cell superresolution microscopy reveals the organization of rna polymerase in the bacterial nucleoid. *PNAS* **2015**, *112*, E4390-E4399.
4. Endesfelder, U.; Finan, K.; Holden, S.J.; Cook, P.R.; Kapanidis, A.N.; Heilemann, M. Multiscale spatial organization of rna polymerase in escherichia coli. *Biophysical journal* **2013**, *105*, 172–181.
5. Lando, D.; Endesfelder, U.; Berger, H.; Subramanian, L.; Dunne, P.D.; McColl, J.; Klenerman, D.; Carr, A.M.; Sauer, M.; Allshire, R.C., *et al.* Quantitative single-molecule microscopy reveals that cenp-a(cnp1) deposition occurs during g2 in fission yeast. *Open biology* **2012**, *2*, 120078.
6. Endesfelder, U.; Malkusch, S.; Flottmann, B.; Mondry, J.; Liguzinski, P.; Verveer, P.J.; Heilemann, M. Chemically induced photoswitching of fluorescent probes--a general concept for super-resolution microscopy. *Molecules* **2011**, *16*, 3106–3118.
7. Datsenko, K.A.; Wanner, B.L. One-step inactivation of chromosomal genes in escherichia coli k-12 using pcr products. *Proceedings of the National Academy of Sciences of the United States of America* **2000**, *97*, 6640–6645.
8. Wang, S.; Moffitt, J.R.; Dempsey, G.T.; Xie, X.S.; Zhuang, X. Characterization and development of photoactivatable fluorescent proteins for single-molecule–based superresolution imaging. *PNAS* **2014**, *111*, 8452–8457.
9. Hayashi, A.; Ding, D.Q.; Tsutsumi, C.; Chikashige, Y.; Masuda, H.; Haraguchi, T.; Hiraoka, Y. Localization of gene products using a chromosomally tagged gfp-fusion library in the fission yeast schizosaccharomyces pombe. *Genes to cells : devoted to molecular & cellular mechanisms* **2009**, *14*, 217–225.
10. Watson, A.T.; Garcia, V.; Bone, N.; Carr, A.M.; Armstrong, J. Gene tagging and gene replacement using recombinase-mediated cassette exchange in schizosaccharomyces pombe. *Gene* **2008**, *407*, 63–74.
11. Berardozzi, R.; Adam, V.; Martins, A.; Bourgeois, D. Arginine 66 controls dark-state formation in green-to-red photoconvertible fluorescent proteins. *Journal of the American Chemical Society* **2016**, *138*, 558–565.
12. Bryksin, A.V.; Matsumura, I. Overlap extension pcr cloning: A simple and reliable way to create recombinant plasmids. *BioTechniques* **2010**, *48*, 463–465.
13. Edelstein, A.; Amodaj, N.; Hoover, K.; Vale, R.; Stuurman, N. Computer control of microscopes using micromanager. *Current protocols in molecular biology / edited by Frederick M. Ausubel [et al.]* **2010**, *Chapter 14*, Unit14 20.
